# Corin Overexpression Reduces Myocardial Infarct Size and Modulates Cardiomyocyte Apoptotic Cell Death

**DOI:** 10.3390/ijms21103456

**Published:** 2020-05-14

**Authors:** Ryan D. Sullivan, Aiilyan K. Houng, Inna P. Gladysheva, Tai-Hwang M. Fan, Ranjana Tripathi, Guy L. Reed, Dong Wang

**Affiliations:** 1 Department of Internal Medicine, University of Arizona College of Medicine-Phoenix, Phoenix, AZ 85004, USA; ryansullivan@arizona.edu (R.D.S.); innagladysheva@arizona.edu (I.P.G.); rtripathi@arizona.edu (R.T.); 2 Department of Medicine, University of Tennessee Health Science Center, Memphis, TN 38163, USA; aiilyan.houng@gmail.com (A.K.H.); tfan1@uthsc.edu (T.-H.M.F.)

**Keywords:** myocardial infarction, corin, apoptosis, Bcl-2 family protein

## Abstract

Altered expression of corin, a cardiac transmembrane serine protease, has been linked to dilated and ischemic cardiomyopathy. However, the potential role of corin in myocardial infarction (MI) is lacking. This study examined the outcomes of MI in wild-type vs. cardiac-specific overexpressed corin transgenic (Corin-Tg) mice during pre-MI, early phase (3, 24, 72 h), and late phase (1, 4 weeks) post-MI. Corin overexpression significantly reduced cardiac cell apoptosis (*p* < 0.001), infarct size (*p* < 0.001), and inhibited cleavage of procaspases 3, 9, and 8 (*p* < 0.05 to *p* < 0.01), as well as altered the expression of Bcl2 family proteins, Bcl-xl, Bcl2 and Bak (*p* < 0.05 to *p* < 0.001) at 24 h post-MI. Overexpressed cardiac corin also significantly modulated heart function (ejection fraction, *p* < 0.0001), lung congestion (lung weight to body weight ratio, *p* < 0.0001), and systemic extracellular water (edema, *p* < 0.05) during late phase post-MI. Overall, cardiac corin overexpression significantly reduced apoptosis, infarct size, and modulated cardiac expression of key members of the apoptotic pathway in early phase post-MI; and led to significant improvement in heart function and reduced congestion in late phase post-MI. These findings suggest that corin may be a useful target to protect the heart from ischemic injury and subsequent post-infarction remodeling.

## 1. Introduction

Myocardial infarction (MI) remains a major public health problem with 550,000 new attacks and 200,000 recurrent attacks per year in the United States [1]. Cardiomyocyte death leads to impaired heart contractility and ischemic cardiomyopathy (ICM) [2]. ICM is the most commonly identified specific cause of dilated cardiomyopathy (DCM) and heart failure (HF) [3,4]. Among those older than 45 who have a first MI, 16% of men and 22% of women will develop HF within 5 years [1]. To improve outcomes for patients post-MI, there is a critical need to identify key factors or new targets that modulate cardiomyocyte death.

Corin is a transmembrane serine protease identified in the heart [5]. An increasing number of basic and clinical studies link alterations in corin expression or corin dysfunction with ICM and DCM. Clinical studies shown that acute decompensated HF is associated with reduced plasma and cardiac corin levels [6,7,8,9]. Dysfunctional corin variants are linked to poor outcomes in patients with systolic HF [10]. Blood corin levels correlate with infarct size and severity in patients post-MI [11,12]. A longitudinal study of experimental DCM shows that cardiac corin levels are an early indicator of cardiomyopathy that correlates with the level of systolic dysfunction even before the onset of HF [13]. Cardiac overexpression of corin decreased cardiac fibrosis, improved HF, and promoted survival in mice with DCM [14].

Apoptosis plays an important role in cardiomyocyte loss and the development of HF in patients with DCM and ICM [15,16]. Since corin reduces the progression of cardiomyopathy and the development of HF in experimental DCM, corin’s effects may be attributable, at least in part, to reduced cardiomyocyte apoptosis. Apoptosis is readily detected following experimental acute MI; apoptotic cell death is a major cause of myocardial damage [17] and affects the size of MI [18].

Findings from one of our previous studies showed that acute MI induces decreases in cardiac corin levels and lower corin levels are inversely correlated with heart function [19]. In the present study, we explored the hypothesis that corin might protect cardiomyocytes from death through inhibition of apoptosis in acute MI. We focused on the intrinsic (or mitochondrial) pathway and the extrinsic pathway, that are well-established in different cardiovascular diseases [20,21]. We show that cardiac corin expression is negatively correlated with infarct size. Cardiac overexpression of corin was associated with modulation of Bcl-2 family proteins, and it markedly reduced apoptosis in acute MI. In addition, consistent with the beneficial modulation at early phase, corin overexpression also leads to a significant improvement in heart function and heart failure progression in chronic phase post-MI. These studies provide the first evidence for a cardioprotective effect of corin in MI, linking it to diminished cardiomyocyte apoptosis and the intrinsic apoptotic pathway.

## 2. Results

### 2.1. Cardiac-Specific Overexpression of Corin Reduces Infarct Size

**Figure 1:**
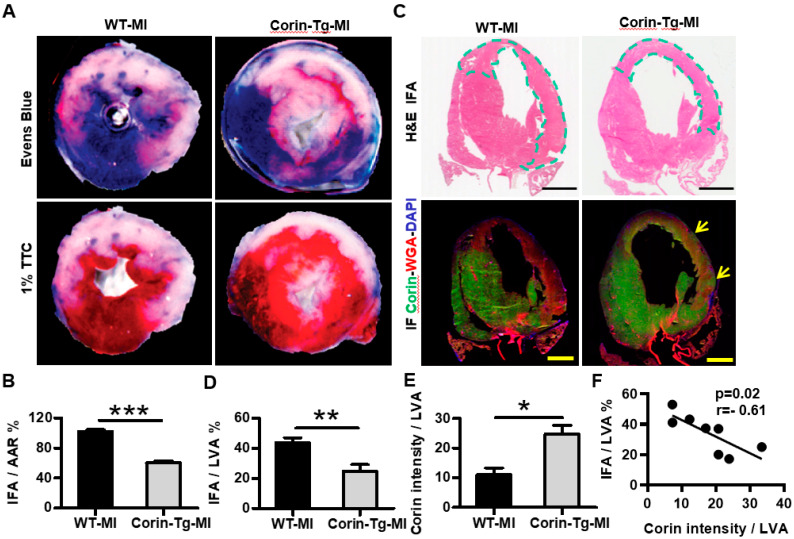
Cardiac-specific overexpression of corin decreases infarct size 24 h post-MI. (**A**) Area at risk for ischemia (AAR; Evans blue staining) and infarct area [IFA; 1% 2,3,5-triphenyl-2H-tetrazolium chloride (TTC)] in representative heart sections from wild-type (WT) & corin-Tg mice 24 h post-MI. (**B**) AAR and IFA were measured in heart sections using Image-Pro Plus software and are shown as the ratio of IFA to AAR. Data represent means ± SE of *n* = 7 mice per group. (**C**) Comparison of the IFA and the area of low corin expression in coronal heart sections. Representative images with H&E staining (top panel) and corin (green) & WGA (red) double-immunofluorescence (IF) staining (DAPI, blue, bottom panel), bar = 100 µm. The IFA was differentiated from non-infarct area by the characteristic eosinophilic staining (highlighted with a dotted line) on H&E sections. In contrast to the WT post-MI heart, there was residual myocardial corin expression (indicated by yellow arrows) in the ischemic area of corin-Tg hearts post-MI (IF sections). (**D**) The IFA (eosinophilic area) and total left ventricular area (LVA) of myocardium were measured using Image-Pro Plus software in H&E stained sections of each heart and the ratio of IFA to LVA was calculated as shown in the bar graph (*n* = 4 per group). (**E**) Corin intensity and LVA were measured using Image-Pro Plus software. The ratios of corin intensity to LVA are shown in the bar graph (*n* = 4 per group). (**F**) Linear regression analysis of IFA/LVA % and corin intensity/LVA in all WT-MI and corin-Tg post-MI groups. Data represent means ± SE for each group. * *p* < 0.05, ** *p* < 0.01, *** *p* < 0.001.

Since cardiac corin overexpression protects against development of DCM [14], we examined whether corin expression may affect infarct size in corin-Tg vs. WT littermates. Myocardial infarct size was assessed 24 h after left anterior descending occlusion by 2,3,5-triphenyl-2H-tetrazolium chloride (TTC) staining of thick heart cross-sections. The infarct area (IFA) normalized to the area at risk (AAR) was significantly smaller in corin-Tg mice than in WT littermates (60.2 ± 2.5% vs. 101.8 ± 2.8%, *p* < 0.01; Figure 1A and 1B). Hematoxylin and eosin (H&E) stained coronal heart sections showed a similar reduction (43%) in the IFA as a percent of the left ventricular area (LVA) in the corin-Tg group vs. WT littermates (24.8 ± 4.4% vs. 43.7 ± 3.3%, *p* < 0.01, Figure 1C top panel and 1D). Cardiac corin protein level was significantly decreased in the infarcted myocardium of both corin–Tg and WT groups (Figure 1C bottom panel and 1E). In WT mice, corin expression was reduced throughout the entire region of infarction, while corin-Tg hearts had areas with preserved corin expression scattered through the infarct region (Figure 1C bottom panel, yellow arrow). Cardiac corin expression was negatively correlated with the infarct size (r = −0.61, *p* < 0.05, Figure 1F).

### 2.2. Cardiac-Specific Overexpression of Corin Significantly Improves Heart Function and Delays Heart Failure Development Post-MI

**Figure 2:**
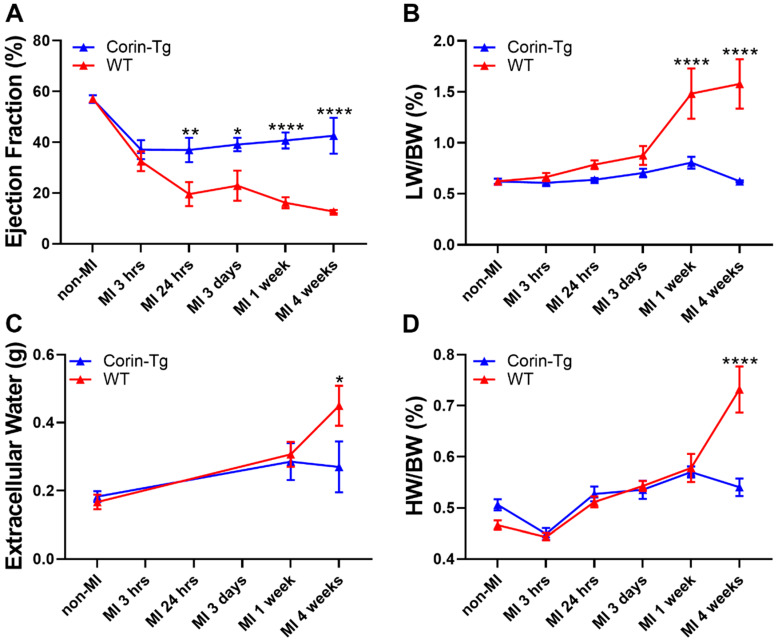
Cardiac-specific overexpression of corin affects heart function in both early and late phase post-MI. (**A**) Dynamic changes in ejection fraction (EF%) (assessed by echocardiography), (**B**) lung weight to body weight ratio (LW/BW%), (**C**) systemic extracellular water (edema) assessed by quantitative magnetic resonance and (**D**) heart weight to body weight ratio (HW/BW%) in mouse groups of WT vs. corin-Tg post-MI. Portions of data from panels A, B, and D (WT group at non-MI, 3 h, 24 h, and 3 days) have been published previously [19] and are included here to reduce animal numbers. Differences between WT and corin-Tg at each study time point were analyzed by 2-way ANOVA with Sidak’s multiple comparisons. Data represent means ± SE of **n** = 3–34 mice per group at each time point. * *p* < 0.05, ** *p* < 0.01, **** *p* < 0.0001 and not significant.

After finding the protective effect of over expressed corin on infarct size during early phase post-MI, we wondered whether such modulations could translate into a beneficial effect on cardiac function and heart failure development during the chronic phase post-MI. We evaluated cardiac function by echocardiography at both early phase (3 h, 24 h, or 3 days) [1] and late phase (1 and 4 weeks) post-MI in WT-MI and corin-Tg-MI groups. Although ejection fraction (EF) dropped in both groups post-MI when compared to non-MI controls, corin-Tg-MI had better EF at most study time points (*p* < 0.01, *p* < 0.05, *p* < 0.0001 and *p* < 0.0001 respectively at 24 h, 3 days, 1 week and 4 weeks) in contrast to WT-MI groups (Figure 2A). A similar trend was also confirmed by fractional shortening (data not shown). Pulmonary congestion, an important clinical sign of heart dysfunction, was more severe in WT-MI groups than in corin-Tg-MI groups evidenced by higher lung weight to body weight ratio (LW/BW, Figure 2B) and systemic extracellular water (edema) assed by quantitative magnetic resonance (Figure 2C). The patterns of LW/BW and systemic extracellular water increases in WT mice supports that pulmonary edema develops prior to pleural effusion in this translational model. There were no significant changes in HW/BW at early time points and only increased at four weeks post-MI (*p* < 0.0001, Figure 2D).

### 2.3. Cardiac-Specific Overexpression of Corin Attenuates Cardiomyocyte Apoptosis Post-MI

**Figure 3:**
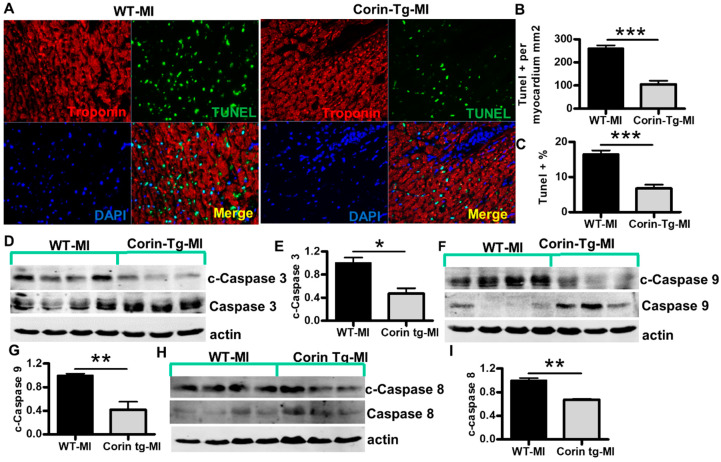
Cardiac-specific overexpression of corin attenuates cardiomyocyte apoptosis 24 h post-MI. (**A**) Representative images of TUNEL staining (green) with troponin (red, cardiac marker) and DAPI-stained nuclei (blue) in the ischemic area of left ventricles (LV) from WT and corin-Tg mouse hearts post-MI (40× magnification). (**B**,**C**) Quantitative digital analysis of TUNEL staining. The total numbers TUNEL-stained cells (TUNEL+) and DAPI-stained nuclei (total cell) in the LV were counted; the total left ventricular area (LVA) was measured at 5× using Image-Pro Plus software. The ratios of TUNEL+ cells per myocardial area (mm2) and the percentages of TUNEL+ cells vs. total cells were calculated. Data represent means ± SE of *n* = 4 mice per group. (**D**,**F**,**H**) Western blot analysis of tissue lysates, prepared using the infarct core and border zone myocardium from corin-Tg-MI (*n* = 3) and WT-MI (*n* = 4) hearts, with antibodies for cleaved (c) caspase 3 (17 kD), caspase 3 (34 kD) c-caspase 9 (37, 39 kD), caspase 9 (37 kD), c-caspase 8 (18 kD), and caspase 8 (55 kD) under reducing conditions. Actin (43 kD) was used as loading control. (**E**,**G**,**I**) Bar graphs represent the densitometry analysis of c-caspase 3, 9 and 8 normalized to actin. Data represent means ± SE of *n* = 3–4 mice per group. * *p* < 0.05, ** *p* < 0.01, *** *p* < 0.001.

After an acute MI, the short and long-term prognosis of patients is highly dependent on infarct size. We wanted to further understand the mechanisms underlying the prevention of cardiomyocyte loss post-MI. Cardiomyocyte apoptosis is considered a major determinant of infarct size in early stage post-MI [18,22]. To determine whether reduced infarct size in corin-Tg mouse hearts was due to fewer cardiomyocytes undergoing apoptosis, we performed TUNEL staining (Figure 3A). The number of TUNEL-positive cardiomyocytes in the LV of the corin-Tg group was much lower than in WT littermates (105.6 ±15.6 cell vs. 260.6 ± 13.2 cell per mm^2^ of myocardium, *p* < 0.001, Figure 3B). There also was a 60% reduction in the percentage of TUNEL-positive cardiomyocytes in corin-Tg vs. WT hearts post-MI (6.7 ± 1.1% vs. 16.6 ± 1.0%, *p* < 0.001, Figure 3C). Most TUNEL-positive cardiomyocytes were located in the border zone and infarct core area, while a minority were present in the remote area. The number of TUNEL-positive myocytes and infarct size were significantly correlated (r = 0.76, *p* < 0.05), suggesting a close relationship (Figure not provided). To evaluate the effect of corin on myocardial apoptosis in acute MI, we assessed the activation/cleavage of major caspases in the intrinsic (or mitochondrial) pathway and the extrinsic pathway [21], including caspase 3, caspase 9 and caspase 8. Consistent with the findings from TUNEL staining, there was a reduction in cleavage of procaspases in corin-Tg vs. WT heart tissue extracts post-MI. Pro-caspase 3 was reduced by 50% (Figure 3D, *p* < 0.05), pro-caspase 9 by 60% (Figure 3F, *p* < 0.01), and pro-caspase 8 by 30% (Figure 3H, *p* < 0.01). Significant myocyte necrosis was also observed in H&E sections of the IFA in corin-Tg and WT hearts post-MI, as indicated by cytoplasmic fragmentation, plasma membrane breakdown, and myocyte vacuolization. There was a suggestion that cell death may be less extensive in ischemic border zone in corin-Tg-MI mice consistent with the smaller infarct size than was observed in WT-MI mice [19].

### 2.4. Corin Overexpression Modulates Bcl-2 Family Proteins in the Infarcted Heart

**Figure 4:**
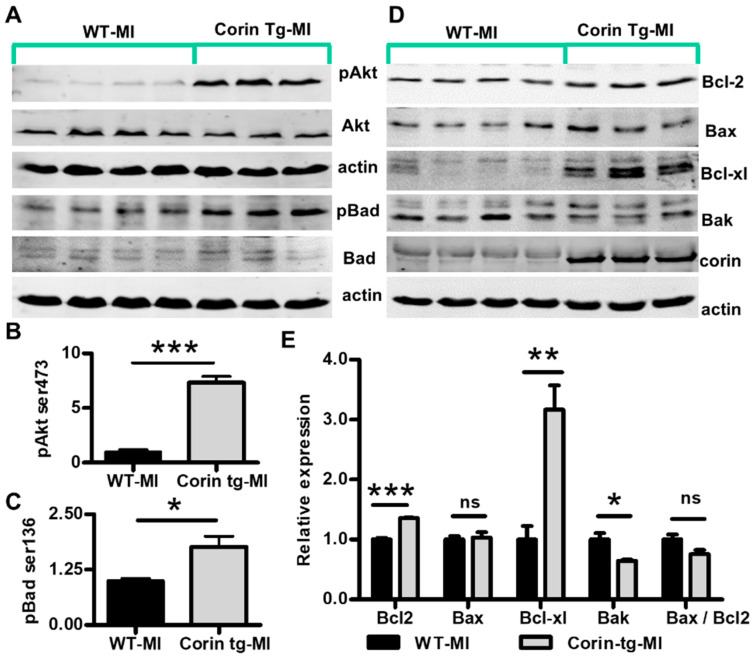
Cardiac corin overexpression modulates activity and expression level of pro-apoptotic and anti-apoptotic proteins in the infarcted heart. (**A**) Expression and phosphorylation of Akt (Ser473) and Bad (Ser136) were assessed by Western-blotting under reduced conditions in post-MI tissues from corin-Tg and WT hearts. Actin was used as loading control. (**B**,**C**) Densitometry analysis of the pAkt (60 kD) and pBad (23 kD) normalized to Akt (60 kD) and Bad (25 kD), respectively. (**D**) Expression of Bcl-2 family proteins, Bcl2, Bax, Bcl-xl, and Bak in corin-Tg and WT hearts post-MI. Cardiac corin (206 kD) overexpression was also confirmed. (**E**) Densitometry analysis of Bcl2 (26 kD), Bax (23 kD), Bcl-xl (30 kD), and Bak (30 kD) expression normalized to actin (43 kD) and, of the Bax/Bcl2 ratio. Heart tissue extracts were prepared as described in Figure 3. Data represent means ± SE of *n* = 3-4 mice per group. * *p* < 0.05, ** *p* < 0.01, *** *p* < 0.001 and ns, not significant.

The intrinsic apoptotic pathway generally plays a more important role after an ischemic insult than the extrinsic pathway [23] and overexpression of corin appeared to have greater effects on caspase 9 cleavage (effector of intrinsic apoptotic pathway) than on caspase 8 (effector of extrinsic pathway) cleavage. Therefore, we focused on the intrinsic apoptotic pathway and examined upstream events that might contribute to the activation of caspase 9. AKT activation is a well-known event reducing apoptotic cardiomyocyte death in response to ischemic injury [24,25]. Phosphorylation of Akt on Ser473, which is critical for full activation of Akt [26], was much higher in corin-Tg than WT hearts post-MI (*p* < 0.01, Figure 4A,B). Bcl-2 family proteins, such as Bad, Bcl2, Bax, Bcl-xl, and Bak, are important modulators of the intrinsic apoptotic pathway [27]. Akt directly inhibits the pro-apoptotic activity of BAD by phosphorylation of Ser136 [28]. Consistent with the enhanced Akt ^Ser473^ phosphorylation, the phosphorylation of Bad^Ser136^ was significantly higher in corin-Tg vs. WT hearts post-MI (Figure 4A,C, *p* < 0.05). Protein expression levels of other important Bcl-2 family proteins, including Bcl2, Bax, Bcl-xl, and Bak, were evaluated in corin-Tg vs. WT hearts post-MI (Figure 4D). Among them, Bcl2 had 1.4-fold increases (*p* < 0.001) while Bax did not show a significant difference (Figure 4E, *p* > 0.05). The Bax/Bcl2 ratio, which determines the susceptibility of cardiac cells to apoptosis [29], tended to be lower in corin-Tg vs. WT hearts post-MI although it did not reach statistical significance (*p* > 0.05, Figure 4E). The expression of the anti-apoptotic molecule Bcl-xl was 3.2-fold upregulated in corin-Tg-MI hearts (*p* < 0.01, Figure 4E). In contrast, Bak expression was downregulated with a 36% drop (*p* < 0.05, Figure 4E).

### 2.5. Corin Overexpression Regulate Bcl-2 Family Proteins under Non-Ischemic Conditions

**Figure 5:**
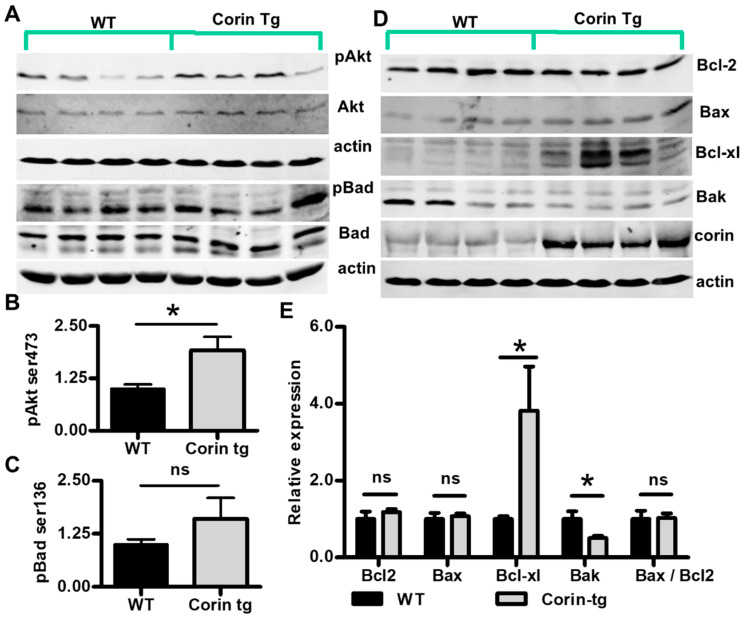
Corin overexpression regulates expression of the Bcl-2 family of proteins expression in non-MI hearts. (**A**) Expression of Akt and Bad, as well as Akt phosphorylation (Ser473) and Bad phosphorylation (Ser136) were assessed by Western-blot under reduced conditions in corin-Tg and WT hearts. Actin (43 kD) was used as a loading control. (**B**,**C**) Densitometry analysis of the pAkt (60 kD) and pBad (23 kD) normalized to Akt (60 kD) and Bad (25 kD), respectively. (**D**) Expression of Bcl-2 family proteins Bcl2, Bax, Bcl-xl, and Bak in corin-Tg and WT mouse hearts. Corin (206 kD) overexpression was also confirmed. (**E**) Densitometry analysis of Bcl2 (26 kD), Bax (23 kD), Bcl-xl (30 kD), and Bak (30 kD) expression normalized to actin (43 kD) and of the Bax/Bcl2 ratio. Heart tissue extracts were prepared as in Figure 3 from WT and corin-Tg mouse hearts; the same antibodies were used for detection as in Figure 4. Data represent means ± SE of *n* = 4 mice per group. * *p* < 0.05 and ns, not significant.

To evaluate if the altered expression of Bcl-2 family proteins shown in corin-Tg mice post-MI was specifically due to ischemia or conditions attributable to corin overexpression itself, we compared expression and phosphorylation of Bcl-2 family proteins in non-MI hearts from corin-Tg vs. WT littermates. Both Akt and Bad showed a trend to enhanced phosphorylation in corin-Tg mice when compared with WT mice (Figure 5A); however, only the increased phosphorylation of Akt was statistically significant (Figure 5B, 1.9 ± 0.3 vs. 1.0 ± 0.1, *p* < 0.05) while the phosphorylation of Bad was not (Figure 5C, 1.6 ± 0.5 vs. 1.0 ± 0.1, *p* > 0.05). Cardiac expression of three Bcl-2 family proteins were identical to acute post-MI conditions (Figure 5D). In corin-Tg hearts vs. WT hearts, Bcl-xl expression was increased 3.8-fold (Figure 5E, *p* < 0.05), while Bak expression was decreased by 50% (Figure 5E, *p* < 0.05), and Bax expression levels remained relatively unchanged (Figure 5E, 1.1 ± 0.1 vs. 1.0 ± 0.2, *p* > 0.05). Bcl2 protein expression level was not modulated by cardiac corin overexpression in the absence of MI (Figure 5E, corin-Tg vs. WT, 1.2 ± 0.1 vs. 1.0 ± 0.2, *p* > 0.05). The Bax/Bcl2 ratio was relatively comparable in both non-MI groups (*p* > 0.05, Figure 5E).

## 3. Discussion

In patients with HF, cardiac and circulating levels of corin decline significantly [6,7,8,9]. Cardiac corin levels are also reduced in various experimental HF models, such as DCM and diabetic cardiomyopathy-related HF [14,30], aortocaval shunt and rapid right ventricular pacing-induced HF [31,32]. Cardiac-selective overexpression of corin modulated HF development and significantly prolonged life in mice with DCM [14]. However, the role of corin in MI and ischemic cardiomyopathy is less well understood. In some studies, circulating corin levels have been noted to be reduced in patients following MI [12] as an indicator of enhanced mortality risk [33], but in other studies low corin levels are associated with smaller infarct size [11]. To better understand corin’s role in this process, we examined cardiac corin transcripts and protein levels in experimental acute MI [19]. We found that corin transcripts and protein expression are specifically reduced in the IFA and larger infarcts are associated with greater reductions of corin expression [19]. Corin appears to have a protective effect in the development of dilated cardiomyopathy-related heart failure [14]. In the present study, cardiac-specific overexpression of corin reduces infarct size and apoptosis, improves heart function and delays heart failure associated with ischemic cardiomyopathy. Corin overexpression is associated with enhanced expression of Bcl-xl and suppression of pro-apoptotic markers. These data provide the first evidence linking corin to cardiomyocyte apoptosis and they begin to define the potential mechanisms that may mediate corin’s protective effect in acute MI.

Corin expression was significantly reduced in the IFA 24 h post-MI. There was an inverse correlation between cardiac corin expression and infarct size of the LV. The reduced infarct size in corin-Tg mouse hearts suggests that corin may protect myocytes against ischemic injury and promote cell survival. In acute MI, apoptosis is an important determinant of cell death and infarct size, although necrosis also plays a role [17,18]. When compared to wild-type litter mates, mice over-expressing cardiac corin showed marked decreases in TUNEL-stained cells and there was also significantly reduced cleaved caspase-3, which is the most important executioner caspase of the final pathway of apoptosis [34]. While this data is the first to link corin to protection against apoptosis, other studies have suggested a potential protective or pro-survival effect of corin. Doi et al. showed that dopaminergic progenitor cells expressing corin survived better in vivo after transplantation [35]. Corin overexpression protects against progressive loss of systolic function, HF, and mortality in experimental dilated cardiomyopathy [14]. In the present study, we also found a similar protective effect of overexpressed corin in MI related ischemic cardiomyopathy and the development of heart failure. After an acute MI, the short and long-term prognosis of patients is highly dependent on infarct size [36]. It is not surprising that corin-Tg-MI group, which showed smaller infarct sizes, also had better heart function through all study time points. Pulmonary congestion is considered to be one of the most important characters of HF [37]. Consistent with EF results, we also observed significant improvement in the magnitude of lung congestion, namely less systemic extracellular water and smaller LW/BW, in corin-Tg group when compared with WT group post-MI. Combined, cardiac corin overexpression was beneficial in preserving heart function and the development of heart failure post-MI.

According to post-MI cardiac proteomic profiling studies, contractile, metabolic, and mitochondrial proteins are downregulated [38,39]. Interestingly, some important apoptosis-related proteins also are downregulated in early stage of MI [40]. For example, the pro-survival protein, apoptotic repressor protein, is significantly downregulated 24 h post-MI [40]. Corin overexpression suppressed ischemia-induced apoptosis and modulated the expression and phosphorylation of Bcl-2 family proteins. Both the intrinsic and extrinsic apoptotic pathways mediate cardiomyocyte death post-MI [41], though the intrinsic pathway may play a more important role in early stage of MI [23]. A working hypothesis of the link between corin and apoptosis is shown in Figure S1 (Data Supplement). Corin overexpression was associated with reduced cleavage of procaspase 9 and procaspase 3 suggesting inhibition of the intrinsic apoptotic pathway [21]. However, corin overexpression was also associated with a 30% reduction in procaspase 8 cleavage, an important event for extrinsic apoptotic pathway activation. More studies are needed in order to demonstrate the possibility of caspase 8 crosstalk with the intrinsic pathway [42]. There was a three-fold increase in Bcl-xl, a member of the Bcl-2 family of proteins, which are upstream controllers of the intrinsic apoptotic pathway [43]. At the same time expression of Bak, a pro-apoptotic factor, was downregulated. Cardiac overexpression of Bcl-xl protects cardiomyocytes from ischemia-reperfusion induced apoptosis and significantly decreases infarct size [44]. Thus, the significant upregulation of cardiac Bcl-xl protein in corin-Tg mice would be expected to reduce ischemia-associated cardiomyocyte apoptosis and infarction [45]. Higher phospho-Bad^ser136^ and increased phospho- Akt^ser473^ levels were also found in corin-Tg vs. WT mice post-MI. Akt is a well-accepted cardiac protective factor that inhibits myocyte apoptosis after ischemic injury [41]. Phosphorylation of Bad at Ser136 is responsible for Akt’s prosurvival effect [46]. These findings provide a preliminary mechanistic explanation for the reduced caspase 9 activation post-MI in hearts overexpressing corin. A similar pattern of Bcl-xl, Bak expression and Bad, Akt phosphorylation was found in non-MI corin-Tg mouse hearts when compared to WT mice hearts, which suggests an enhanced pro-survival potential at baseline prior to MI.

Although we have shown that corin overexpression decreases cardiac apoptosis and delays the decline in systolic function and onset of HF, more detailed mechanistic studies are needed to define the functional effect of corin in MI and ischemic cardiomyopathy. For example, since atrial natriuretic peptide (ANP), which is activated by corin cleavage, has been shown to have diverse effects on apoptosis, it will be key to determine whether corin’s anti-apoptotic effect is ANP-dependent or -independent. Additional work is needed to better define the mechanisms through which corin affects apoptosis and to carefully assess its effects on necrotic cell death. Clinically, measurements of circulating corin level post-MI appear to provide conflicting results [11,12,33]; thus, from a translational perspective, to better understand the significance of circulating corin levels, more work is needed to establish the relationship between cardiac corin expression and the levels of circulating, immunoreactive corin detected by current assays. Additionally, post-infarction remodeling, such as infarct size extension, adaptive cardiomyocyte hypertrophy, progressive ventricular fibrosis, chamber dilatation, and systolic dysfunction post-MI is considered as the primary target to improve outcomes for MI patients. Therefore, it will be important to determine the effect of corin on these aspects in the development of ventricular remodeling and ischemic cardiomyopathy during chronic recovery from acute MI. In particular, both ANP and B-type natriuretic peptide appear to inhibit cardiac fibrosis by modulating renin-angiotensin-aldosterone system signaling [47]. We previously showed corin’s anti-fibrotic effect in DCM mouse model [14]. It would be interesting to know whether corin has similar anti-fibrotic effect post-MI through the corin-ANP/BNP pathway.

In conclusion, this study provides the first evidence that corin overexpression reduces apoptosis and infarction in an in vivo model of MI with translational relevance to human cardiovascular disease. Much is yet to be learned, but these data suggest that modulation of corin activity and, assessment of corin expression, may prove to have therapeutic and prognostic value in ischemic heart disease.

## 4. Materials and Methods

### 4.1. Mice

Corin cardiac-transgenic (corin-Tg) mice were produced in our laboratory using the α-myosin heavy chain promoter in a CD1 background [14]. Wild type (WT) littermate mice served as controls throughout the studies. Mice were housed in accordance with institutional and national regulatory standards as outlined in the Guide for the Care and Use of Laboratory Animals 8th Edition. The microenvironment consisted of Optimice (Animal Care Systems, Inc., Centennial, CO, USA) ventilated rack systems with ad lib access to hyperchlorinated facility water and fixed formula maintenance diet Teklad 7912 (Envigo, Madison, WI, USA). All animal procedures were approved by the Institutional Animal Care and Use Committee at the University of Tennessee Health Science Center (Protocol 14-083.0, approved 09/23/2015).

### 4.2. Myocardial Infarction Model

Myocardial infarction (MI) was induced by left anterior descending (LAD) coronary artery ligation with minor modifications, as previously described [48]. Anesthesia induction was achieved using 3% isoflurane in oxygen, eyes were lubricated and buprenorphine (0.1 mg/kg, SC) was administered for analgesia. Mice were then intubated with a 20G × 1 inch catheter. Maintenance anesthesia at 1.5–2% isoflurane in oxygen was delivered via a rodent ventilator (Harvard Apparatus, Boston, MA, USA) set at 120 breaths per minute and 0.2 mL stroke volume. Chest fur was removed with clippers and then skin aseptically prepared with alternating scrubbing of betadine and alcohol soaked gauze. The mouse was secured to a heated surgical platform (36–37 °C) and surgical drape applied. A thoracotomy (left third intercostal space) was performed using scissors and a retractor placed to maintain exposure. The pericardium was incised, and a saline soaked cotton ball was used to position the heart for visualization of the LAD. Ligation was placed 2–3 mm from the origin using 7-0 silk suture and ischemia of the left ventricle (LV) confirmed visually. The cotton ball was removed, lungs were fully inflated, and the thoracotomy was closed using absorbable suture for the muscle and nylon suture for the skin. Isoflurane was discontinued and mice were ventilated on 100% oxygen until fully recovered. Mice were returned to a fresh housing box with thermal support. At each study time point after MI (3 h, 24 h, 3 days, 1 week, and 4 weeks), tissues and blood were harvested and subjected to analysis.

To determine the myocardial area at risk (AAR) for infarction, mice were perfused with 2% Evans blue dye (Sigma-Aldrich, Saint Louis, MO, USA) via the right jugular vein while heavily anesthetized with 5% isoflurane in oxygen. The LVs (septum and free wall) were isolated from the excised hearts and cut into three 2-mm transverse slices distal to the suture. Each slice was weighted and digitally photographed. Next, slices were stained with 1% 2,3,5-triphenyl-2H-tetrazolium chloride (Sigma-Aldrich, Saint Louis, MO, USA) [19]. The infarcted area (IFA, white) and the healthy myocardium (dark red) were digitally photographed. Finally, all slices were fixed in 10% buffered formalin (Thermo Fisher Scientific, Middletown, VA, USA). Two blinded observers quantified the digital images for IFA, AAR, and total left ventricle area (LVA) using Image Pro Plus 6.2 (Media Cybernetics, Bethesda, MD, USA), as described previously [19].

### 4.3. Echocardiography

Cardiac function was assessed for all mice at baseline (pre-MI) and at post-MI study endpoints for each group as previously described [13,14,19,49,50,51,52] by an experienced and blinded operator. Briefly, mice were anesthetized with 3–5% isoflurane in O2 for induction, followed by 1.5–2% isoflurane in O2 for maintenance. Body temperature was maintained at 37 ± 1 °C and heart rate 450 ± 50 BPM. If necessary, fur was removed with depilatory cream. Transthoracic standard images, utilizing parasternal long-axis and short-axis views (Vevo 2100, FUJIFILM VisualSonics, Toronto, ON, Canada), were recorded in B-mode and M-mode using a 30 MHz transducer for post-procedural analysis using Vevo Lab (3.1.0, FUJIFILM VisualSonics, Inc., Toronto, ON, Canada) software with standard equations.

### 4.4. Quantitative Magnetic Resonance

Mouse body composition was measured longitudinally from baseline to study endpoints as previously described [49] using quantitative magnetic resonance (EchoMRI 4-in-1, EchoMRI Inc., Houston, TX, USA). Mice were weighed (Scout Pro SP401, Ohaus Corporation, Pine Brook, NJ, USA), then secured in the provided restraint tube. The tube was inserted into the corresponding port on the machine for measurement recording (approximately 90 s). Following completion, the fully conscious mice were returned to their housing box.

### 4.5. Heart Tissue Lysate Preparation and Western Blot Analysis

Isolated LVs were sliced into three 2-mm cross sections from the level of ligation site. The top two sections were further dissected into two parts—the IFA plus border zone and the remote area. For the non-MI group, myocardium from similar location was prepared in the same way. Different parts of the heart were snap-frozen with liquid nitrogen and kept in separate tubes at −80 °C for downstream applications. About 20–25 mg heart tissue from the first part (IFA plus border zone) were used for preparing protein lysate in lysis buffer (20 mmol/L HEPES, pH 7.2, 25 mmol/L NaCl, 2 mmol/L EGTA and 1% SDS). Protease inhibitor cocktail and phosphatase inhibitor cocktail (biotool.com) were added according to the product instructions. After testing the protein concentration using Pierce™ BCA Protein Assay Kit (Thermo Fisher Scientific #23225), either 50 ug or 100 ug aliquots were made for each sample and kept in −20 °C. Western blot analysis was performed under reduced condition and immunoblot with following antibodies: cleaved caspase-9 (Asp353), caspase-9, cleaved caspase-3 (Asp175), cleaved caspase-8 (Asp387), phospho-Akt (Ser473), phospho-Bad (Ser136) were from Cell Signaling; caspase-8 (1G12, Enzo Life Science Inc., Farmingdale, NY, USA); caspase-3, Akt, Bcl2, Bax, Bad, Bak, actin were from Santa Cruz Biotechnology, Inc. (Dallas, TX, USA); rabbit polyclonal anti-corin [13,14]. All antibody source information, species, and dilutions used can be found in Table 1. Membranes were then labeled with fluorescent secondary antibodies, including goat anti-mouse, goat anti-rabbit, or donkey anti-goat antibody, and visualized using the Odyssey system (Licor Biosciences, Lincoln, NE, USA). Some of these membranes were then stripped with the Restore™ PLUS Western Blot Stripping Buffer (Thermo Fisher Scientific #46430) to re-probe with other primary antibodies. Protein loading was normalized by α-actin. Densitometry quantification was done using Image J software (NIH). Briefly, an area of interest was selected around the band(s) and the same selection box was applied to all bands. Similar to single bands, multiple bands were selected as a single area of interest and summated by the program to provide a total signal for that protein. Examples of multiple band proteins include c-Caspase 9 (Figure 3F) 2 bands; Bad (Figure 4A and Figure 5A) 2 bands; Bcl-xl (Figure 4D and Figure 5D) 3 bands; Bak (Figure 4D and Figure 5D) 2 bands.

### 4.6. Immunohistological Staining and Analysis

Frozen mouse hearts were embedded in optimal cutting temperature (O.C.T.) compound (Sakura Finetek USA, Inc., Torrance, CA, USA) and cut into 5 µm coronal cryosections. Myocardial infarction was assessed with H&E stained slides, imaged with digital scanner (Aperio ScanScope, Vista, CA, USA). LV IFA was denoted by characteristic eosinophilic staining compared to the non-IFA. Image-Pro Plus was used to measure Total LVA and IFA, which was reported as a ratio (IFA/LVA). Corin was stained as described previously [14,51] using our lab‘s internal rabbit anti-corin antibody [53,54] to measure expression. The apoptotic cells were determined by double-immunofluorescence staining with TUNEL staining (In Situ Cell Death Detection Kit, Fluorescein. Roche Applied Science, Penzberg, Germany) and cardiac marker (troponin, # ab56357, Abcam, Cambridge, MA, USA). Slides were scanned with the Aperio image fluorescence scanner (Aperio ScanScope CS2, Vista, CA, USA) and images were taken using ImageScope software (MAN-0001, revision G) at 5× and 40× magnification. To evaluate cardiac corin level, total gross fluorescence of LV and total LVA of each heart were measured using Image Pro Plus 6.2 (Media Cybernetics, Bethesda, MD, USA). The result was expressed as ratio of total fluorescence intensity to LVA. For the apoptotic assay, the numbers of total TUNEL stained cells and DAPI stained nuclei in the LV, as well as the total myocardial area of LV were measured at 5× magnification using Image-Pro Plus software, and the number of TUNEL+ cells per area of the myocardium (mm^2^) and the percentage of TUNEL+ cells were calculated.

### 4.7. Statistics

Differences between groups were analyzed by unpaired t-test or two-way ANOVA with Sidak’s multiple comparisons test. Data reported as mean ± standard error (SE). Statistical associations between two variables were calculated using Pearson’s correlation coefficient (r). All data were analyzed using Prism 8 (GraphPad Software, San Diego, CA, USA). Differences were considered significant if *p* < 0.05. Group sizes (*n*) are provided in figure legends.

## Figures and Tables

**Table 1:** Antibody List.

Target	Company	Cat #	Host Species	Clonality	Dilution
**cleaved caspase-3**	Cell Signaling	9661s	rabbit	polyclonal	1:1000
**caspase-3**	Santa Cruz Biotechnology	sc-7148	rabbit	polyclonal	1:400
**cleaved caspase-9**	Cell Signaling	9509s	rabbit	polyclonal	1:1000
**caspase-9**	Cell Signaling	9508s	mouse	monoclonal	1:1000
**cleaved caspase-8**	Cell Signaling	8592s	rabbit	monoclonal	1:1000
**caspase-8**	Enzo Life Science	ALX-804-447-C100	Rat	monoclonal	1:500
**actin**	Santa Cruz Biotechnology	sc-1616	Goat	polyclonal	1:500
**phospho-Akt (Ser473)**	Cell Signaling	4060s	rabbit	monoclonal	1:1000
**Akt1/2/3**	Santa Cruz Biotechnology	sc-8312	rabbit	polyclonal	1:500
**phospho-Bad (Ser136)**	Cell Signaling	4366s	rabbit	monoclonal	1:500
**bad**	Santa Cruz Biotechnology	sc-943	rabbit	polyclonal	1:500
**Bcl2**	Santa Cruz Biotechnology	sc-492	rabbit	polyclonal	1:500
**Bax**	Santa Cruz Biotechnology	sc-493	rabbit	polyclonal	1:500
**Bak**	Santa Cruz Biotechnology	sc-832	rabbit	polyclonal	1:500
**Bcl-xL**	Santa Cruz Biotechnology	sc-8392	mouse	monoclonal	1:500
**corin**	Lab Generated [53,54]	N/A	rabbit	polyclonal	1:7000

Not applicable (N/A).

## Supplementary Materials

Supplementary materials can be found at https://www.mdpi.com/1422-0067/21/10/3456/s1. Figure S1. Potential mechanisms responsible for the protective effect of cardiac corin overexpression on reduction of cardiomyocyte death and infarct size in acute MI.

## Abbreviations

MI	Myocardial infarction
ICM	Ischemic cardiomyopathy
DCM	Dilated cardiomyopathy
HF	Heart failure
WT	Wild-type
Corin-Tg	Corin transgenic
EF	Ejection fraction
FS	Fractional shortening
LW/BW	Lung weight to body weight ratio
HW/BW	Heart weight to body weight ratio
TCC	2,3,5-triphenyltetrazolium chloride
H&E	Hematoxylin and eosin
AAR	Area at risk for ischemia
IFA	Infarct area
IF	Immunofluorescence
LV	Left ventricle
LVA	Left ventricular area
ANP	Atrial natriuretic peptide
