# Supplementary material for: Corin Overexpression Reduces Myocardial Infarct Size and Modulates Cardiomyocyte Apoptotic Cell Death
Source: Int J Mol Sci. 2020 May 14;21(10):3456. doi: 10.3390/ijms21103456 (PMC7278931; doi:10.3390/ijms21103456)
Supplement: Supplementary file 1 [file ijms-21-03456-s001.pdf]

**Supplementary Materials:** Supplementary materials can be found at http://www.mdpi.com/1422-0067/21/10/3456/s1.

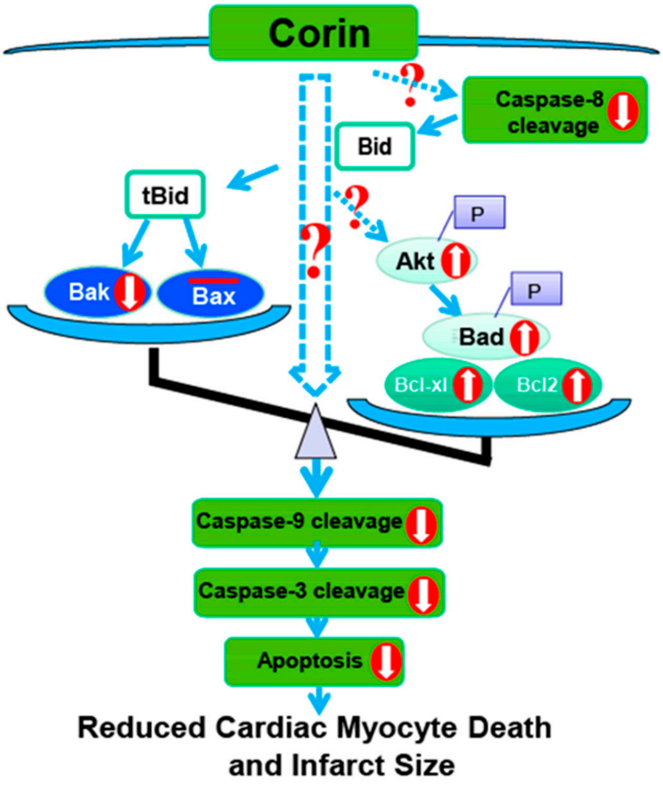


**Figure S1. Potential mechanisms responsible for the protective effect of cardiac corin overexpression on reduction of cardiomyocyte death and infarct size in acute MI.** Corin may inhibit the intrinsic apoptotic pathway by regulating Bcl-2 family proteins. For example, corin overexpression upregulated phosphorylation of Bad and expression of Bcl-xl, Bcl2. At the same time the expression of Bak was downregulated, shifting the balance from a pro-apoptotic to an anti-apoptotic state, and diminishing caspase 9 and caspase 3 cleavage. Alternatively, corin overexpression may regulate the activation of caspase 8 and the Bcl-2 family proteins through cross-talk between the extrinsic and intrinsic pathways, followed by reduced cardiomyocyte death and infarct size. Solid arrows show the well-established apoptotic pathway; dotted arrows and question marks (?) indicate potential, but unknown mechanisms or pathways; the green or blue-shaded text boxes indicate molecules probed in the present study; the white filled boxes indicate untested, but well-accepted effectors. The "↑", "–" and "↓" indicate upregulated, unchanged and downregulated effects, respectively, noted in this study.
